# Remnant Cholesterol and Cardiovascular Risk in Adults With Type 1 Diabetes: A Nested Case–Control Study

**DOI:** 10.1002/edm2.70114

**Published:** 2025-10-08

**Authors:** Fernando Sebastian‐Valles, Iñigo Hernando‐Alday, Luis Eduardo Lander Lobariñas, Maria Luisa Palacios Berraquero, Jon Garai‐Hierro, Gisela Liz Roman‐Gomez, Álvaro Montes Muñiz, Victor Navas‐Moreno, Purificación de Martinez Icaya, Juan José Raposo‐López, Miguel Antonio Sampedro‐Nuñez, Carmen González‐Ávila, Jose Alfonso Arranz‐Martín, Mónica Marazuela

**Affiliations:** ^1^ Department of Endocrinology and Nutrition, Hospital Universitario de La Princesa, Instituto de Investigación Sanitaria de La Princesa Universidad Autónoma de Madrid Madrid Spain; ^2^ Department of Endocrinology and Nutrition Hospital Universitario Basurto Bilbao Spain; ^3^ Department of Endocrinology and Nutrition Hospital Universitario Severo Ochoa Madrid Spain; ^4^ Department of Hematology Hospital Universitario 12 de Octubre Madrid Spain; ^5^ Department of Cardiology Hospital Universitario de La Princesa Madrid Spain; ^6^ Department of Neurology Hospital Universitario Infanta Elena Valdemoro Spain

**Keywords:** cardiovascular disease, remnant cholesterol, type 1 diabetes

## Abstract

**Background:**

Despite advancements in therapeutic strategies, adults with type 1 diabetes (T1D) remain at high risk of cardiovascular disease (CVD). Traditional lipid parameters may not fully account for this residual risk. Remnant cholesterol—found in triglyceride‐rich lipoproteins such as VLDL and IDL—has emerged as a potential contributor to atherogenesis but has not been extensively studied in T1D.

**Methods:**

We conducted a nested case–control study within a multicentre Spanish cohort of 2187 adults with T1D. A total of 88 cases with a first CVD event (2010–2024) were matched 1:1 to controls by age, sex, diabetes duration, HbA1c, smoking, hypertension and retinopathy. Remnant cholesterol was calculated as total cholesterol minus HDL‐C and LDL‐C. Multivariable conditional logistic regression was used to assess associations with CVD. A stratified analysis by LDL‐C quartiles was also performed to explore potential effect modification.

**Results:**

The mean age of participants was 59.9 ± 12.1 years, 34.7% were female, the median duration of diabetes was 27.9 ± 13.3 years and mean HbA1c was 7.9%±1.3%. Remnant cholesterol levels in the highest quartile (> 28 mg/dL) were independently associated with increased risk of CVD (OR = 4.50; 95% CI: 1.34–15.08; *p* = 0.015). This association was evident only among individuals with LDL‐C ≥ 100 mg/dL, while no significant relationship was observed in those with LDL‐C < 100 mg/dL. A linear trend across LDL‐C strata further supported a dose–response relationship. HDL‐C < 45 mg/dL and triglycerides in the highest quartile were also associated with increased CVD risk. Notably, most participants did not achieve LDL‐C targets and 41.4% were untreated.

**Conclusions:**

Elevated remnant cholesterol is an independent predictor of cardiovascular disease in adults with T1D, particularly in those with suboptimally controlled LDL cholesterol. These findings highlight the importance of achieving LDL‐C treatment goals and considering remnant cholesterol in cardiovascular risk assessment, supporting the need for targeted lipid‐lowering strategies in T1D.

## Graphical abstract

In adults with type 1 diabetes, elevated remnant cholesterol was independently associated with cardiovascular disease, particularly in those with LDL‐C ≥ 100 mg/dL. Achieving LDL‐C targets is essential to minimise residual cardiovascular risk and underscores the need to consider remnant cholesterol in risk assessment.

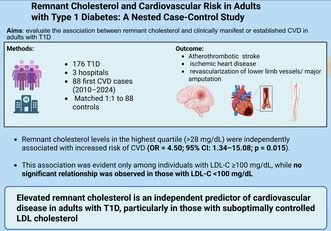

## 1 Introduction

Type 1 diabetes mellitus (T1D) is a chronic disease that predominantly affects young individuals and has shown a rising incidence across Europe in recent decades [1]. In 2019, an estimated 1.5 million people under the age of 20 were living with T1D worldwide [2] and diabetes‐related deaths (types 1 and 2 combined) in individuals under 25 years totalled approximately 16,300, with more than 70% attributed to T1D [3]. Despite advances in therapeutic management, a substantial residual risk of cardiovascular disease (CVD) persists in this [4, 5] population, underscoring the need to identify nontraditional and underrecognised contributors to atherogenesis [6].

In recent years, remnant cholesterol—the cholesterol content of triglyceride‐rich lipoproteins, including very‐low‐density lipoproteins (VLDL) and intermediate‐density lipoproteins (IDL)—has emerged as a potential key mediator of residual cardiovascular risk [7, 8]. Unlike LDL cholesterol, which is the primary target of lipid‐lowering therapies, remnant cholesterol reflects atherogenic particles that carry both cholesterol and proinflammatory components and may contribute directly to plaque formation and progression [8, 9, 10, 11]. In the general population, elevated remnant cholesterol has been independently associated with increased risk of ischaemic heart disease and all‐cause mortality, even after adjustment for traditional lipid parameters [12].

However, evidence regarding the role of remnant cholesterol in individuals with T1D remains limited. This is particularly relevant given the characteristic alterations in lipid metabolism observed in T1D, such as insulin‐deficient hepatic lipoprotein regulation, altered LDL particle morphology, increased systemic inflammation and frequently preserved or elevated HDL cholesterol levels—all of which may mask underlying atherogenic risk [13, 14, 15]. Moreover, standard lipid panels often fail to capture the full burden of atherogenic remnant particles [16], which may partially explain the persistent cardiovascular risk seen in this population despite seemingly favourable lipid profiles.

This study aims to evaluate the association between remnant cholesterol and clinically manifest or established CVD in adults with T1D, using a nested case–control analysis within a well‐characterised multicentre cohort. By focusing specifically on remnant cholesterol and its contribution to cardiovascular events, we seek to improve understanding of nontraditional lipid‐related risk factors in T1D and to explore their potential utility in refining risk prediction and preventive strategies.

## 2 Methods

### 2.1 Study design, inclusion and exclusion criteria

We conducted a retrospective case–control study including adult patients with T1D from three Spanish centres who experienced a first CVD event between 2010 and 2024 and were alive at the time of study inclusion. The inclusion criteria were a confirmed diagnosis of T1D under regular follow‐up at one of the three participating centres and the occurrence of a CVD event. The definition of CVD encompasses chronic ischaemic heart disease (diagnosed by stress test or cardiac catheterisation), acute myocardial infarction, atherothrombotic stroke, need for revascularisation of lower limb vessels and major amputation. Exclusion criteria included other types of diabetes, absence of a complete lipid profile in the six months prior to the event, amputations due to diabetic foot and patients with angina from valvular causes or embolic stroke. This study adhered to the Strengthening the Reporting of Observational Studies in Epidemiology (STROBE) guidelines [17] and was approved by the Ethics Committee of each Hospital (Study number: 5601‐9‐05‐24, act 09/24), in accordance with the principles of the Declaration of Helsinki. The local ethics committees, due to the retrospective nature of the study and the use of de‐identified clinical data, waived the requirement for informed consent.

### 2.2 Data collection

Sociodemographic information, clinical data, laboratory tests and medications used for T1D were collected from electronic medical records. Smoking was defined as the daily use of at least one cigarette, cigar or pipe (excluding electronic cigarettes). Variables collected included sex, age, type of diabetes, diabetes duration, body mass index (BMI), smoking behaviour, insulin dose and the Spanish deprivation index [18] as a proxy for socioeconomic status, as previously done by similar studies [19, 20, 21]. Data on nephropathy, retinopathy, ischaemic heart disease and non‐fatal ischaemic stroke were also collected. Nephropathy and retinopathy were classified according to international standards [22, 23], with nephropathy defined as an estimated glomerular filtration rate (eGFR) below 60 ml/min 1.73 m^2^. Lipid‐lowering therapy was categorised according to the potency of LDL cholesterol reduction, as recommended by clinical guidelines [24].

### 2.3 Determination of lipid profiles

Cholesterol was measured using an enzymatic method (Alinity C Cholesterol Reagent Kit, Abbott) and HbA1c was determined by high‐performance liquid chromatography (ADAMS A1c HA8180 V, ARKRAY). HbA1c values were obtained at the time of the cardiovascular event and averaged from the three most recent determinations prior to the event. Plasma samples were collected after an overnight fast and all biochemical analyses were performed in local laboratories. Plasma glucose was measured using the glucose oxidase method; total cholesterol via the esterase–oxidase–peroxidase method; triglycerides using the glycerol phosphate oxidase–peroxidase method and HDL cholesterol (HDL‐C) was measured directly following precipitation with phosphotungstic acid and magnesium chloride. When triglyceride levels were below 300 mg/dL, LDL cholesterol was calculated using the Friedewald formula. Remnant cholesterol was estimated as total cholesterol minus LDL‐C and HDL‐C. Lipid variables were categorised into quartiles, in accordance with previous studies [7, 25].

### 2.4 Statistical Analysis

The normality of continuous variables was assessed using the Kolmogorov–Smirnov test and visual inspection of normal probability plots. Differences in continuous variables between cases and controls were evaluated using the Student's t‐test for normally distributed data or the Wilcoxon rank‐sum test for non‐normally distributed variables. Categorical variables were compared using the chi‐square test.

To address potential confounding due to the observational design, each case was matched 1:1 to a control using nearest‐neighbour propensity score matching without replacement (caliper width = 0.05). Matching variables included age, sex, duration of T1D, hypertension, diabetic retinopathy, smoking status and HbA1c, which are established cardiovascular risk factors [26]. Unadjusted associations were analysed using conditional logistic regression for matched data. In addition to the variables included in the propensity score matching, the following were evaluated as potential confounders: insulin/kg, nephropathy, BMI, no lipid‐lowering therapy and high‐intensity statin therapy. To assess their role, we used the user‐written confound [27] command in Stata, which identifies as confounders those covariates whose exclusion from the model results in a ≥ 10% change in the effect estimate of the main exposure (remnant cholesterol quartiles). A stratified analysis was also performed based on LDL cholesterol quartiles, as previously described in other studies and conditional logistic regression was repeated within LDL subgroups (< 100 mg/dL and ≥ 100 mg/dL) to evaluate potential effect modification [7]. The threshold for remnant cholesterol concentration in relation to cardiovascular risk was set at 30 mg/dL, consistent with prior evidence [28]. All statistical analyses were conducted using Stata 17.0 BE (StataCorp LLC, College Station, TX, USA). A two‐sided *p*‐value < 0.05 was considered statistically significant.

## 3 Results

### 3.1 Study Population

**FIGURE 1:**
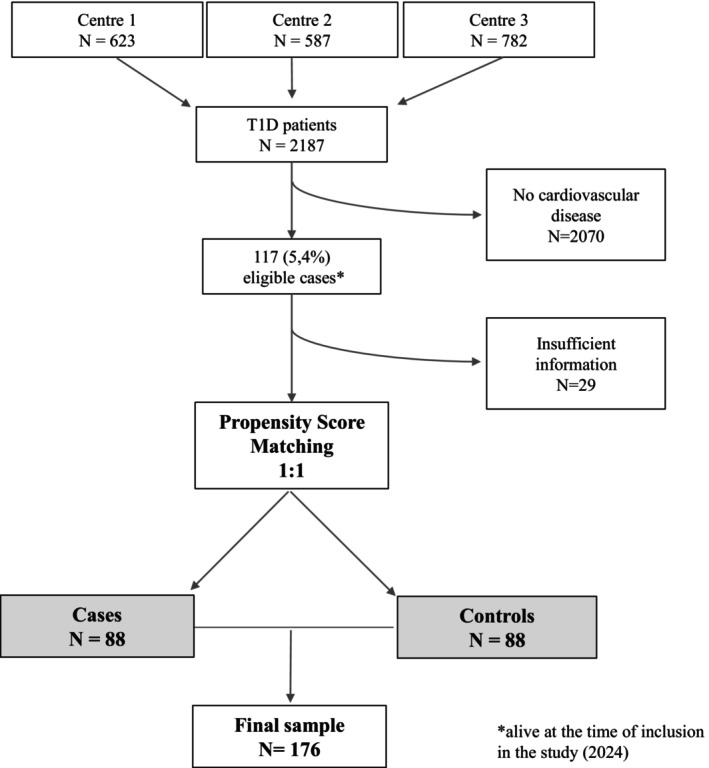
Patient selection flow chart T1D: Type 1 Diabetes.

After applying the aforementioned inclusion and exclusion criteria, 117 (5.4%) patients from a multicentre cohort of 2187 individuals with T1D were included in the study. Of these, 29 patients lacked sufficient clinical data at the time of their cardiovascular event, resulting in a final case sample of 88 subjects. Propensity score matching did not exclude any cases, allowing for a final sample of 176 individuals (88 cases and 88 controls). Among the 88 cases, 60 (68.1%) were ischaemic heart disease, 14 (15.9%) were atherothrombotic stroke and 14 (15.9%) were major lower limb amputations or required revascularisation of lower limb vessels. Figure 1 presents the patient recruitment flowchart.

### 3.2 Baseline Characteristics

The mean age of participants was 59.9 ± 12.1 years, with 34.7% being female. The average duration of diabetes was 27.9 ± 13.3 years and the mean HbA1c was 7.9%± 1.3%. Hypertension was present in 62.5% of participants and 26.7% were current smokers. Diabetic retinopathy and nephropathy were present in 60.2% and 27.8% of the sample, respectively. There were no significant between‐group differences in age, sex, BMI, diabetes duration, HbA1c, insulin dose or socioeconomic deprivation score (Table 1).

**TABLE 1:** Population characteristics.

	Obs, *n* = 176	Control, *N* = 88	Cases, *N* = 88	*P* value
Age (years)	59.9 ± 12.12	59.8 ± 12.9	59.9 ± 11.4	0.854
Sex (Women)	61 (34.7%)	29 (32.9%)	32 (36.7%)	0.635
Hypertension	110 (62.5%)	55 (62.5%)	55 (62.5%)	1.000
Smoking habit	47 (26.7%)	21 (23.9%)	26 (29.6%)	0.394
BMI (Kg/m^2^)	26.7 ± 4.2	26.8 ± 4.10	26.6 ± 4.4	0.687
Duration of diabetes (years)	27.89 ± 13.3	28.2 ± 13.50	27.6 ± 13.4	0.761
Diabetic retinopathy (%)	106 (60.2%)	48 (54.6%)	58 (65.9%)	0.124
HbA1c (%)	7.93 ± 1.32	7.80 ± 1.04	8.07 ± 1.53	0.158
Privation Index	‐0.754 ± 0.872	‐0.948 ± 0.872	‐0.727 ± 0.785	0.080
Lipid‐lowering drugs				
Non‐lipid‐lowering drugs	73 (41.5%)	47 (53.4%)	26 (29.6%)	0.001
Low‐potency	5 (2.8%)	2 (2.3%)	3 (3.4%)	0.650
Moderate‐potency	51 (29.0%)	23 (26.1%)	28 (31.8%)	0.406
High‐potency	47 (26.7%)	16 (18.2%)	31 (35.2%)	0.011
Diabetic nephropathy	49 (27.8%)	16 (18.2%)	33 (37.5%)	0.004
Previous average HbA1c (%)	7.92 ± 1.22	7.80 ± 0.98	8.04 ± 1.41	0.179
Insulin dose IU/kg/day	0.63 ± 0.27	0.60 ± 0.235	0.67 ± 0.29	0.107
Lipid outcomes				
Total cholesterol (mg/dL)	174 ± 41	184 ± 34	164 ± 46	0.002
LDL (mg/dL)	95 ± 34	101 ± 27	89 ± 39	0.012
HDL (mg/dL)	55 ± 17	61 ± 15	49 ± 18	< 0.001
Triglycerides (mg/dL)	113 ± 80	92 ± 40	133 ± 102	< 0.001
Remnant cholesterol (mg/dL)	24 ± 15	20 ± 13	26 ± 17	0.019
Excess remnant cholesterol	40 (22.7%)	13 (14.7%)	27 (30.7%)	0.012

Abbreviations: BMI, body mass index; HbA1c, glycated haemoglobin excess remnant cholesterol was defined as > 30 mg/dL of remnant cholesterol in plasma.

However, diabetic nephropathy was significantly more common in cases (37.5%) than in controls (18.2%, p = 0.004). Similarly, lipid‐lowering therapy use differed between groups: 53.4% of controls were not receiving any lipid‐lowering treatment versus only 29.6% of cases (*p* = 0.001), while high‐potency statin use was more frequent among cases (35.2%) than in controls (18.2%, *p* = 0.011).

### 3.3 Lipid Profile and Cardiovascular Disease

Cases exhibited lower levels of total, LDL and HDL cholesterol and higher triglycerides and remnant cholesterol. Notably, elevated RC (> 30 mg/dL) was more frequent among cases (30.7%) than controls (14.7%, *p* = 0.012) (Table 1).

**FIGURE 2:**
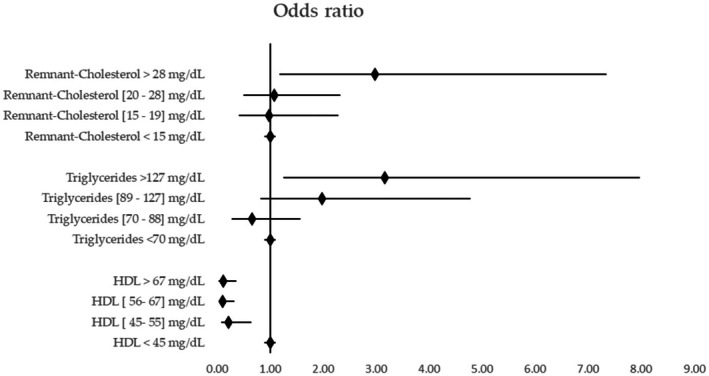
Lipid fractions and risk of cardiovascular disease. A consistent association is observed between the upper quartile of remaining cholesterol and triglycerides and the lower quartile of HDL with cardiovascular disease. Remnant cholesterol: <15 mg/dL (Ref), 15–19 mg/dL: OR = 1.26 (95% CI: 0.46–3.46), 20–28 mg/dL: OR = 1.30 (95% CI: 0.50–3.36), >28 mg/dL: OR = 2.94 (95% CI: 1.38–7.34). Triglycerides:<70 mg/dL (Ref), 70–88 mg/dL: OR = 0.93 (95% CI: 0.37–2.37), 89–127 mg/dL: OR = 2.33 (95% CI: 0.88–6.19), >127 mg/dL: OR = 3.16 (95% CI: 1.16–8.58). HDL cholesterol:<45 mg/dL (Ref), 45–55 mg/dL: OR = 0.23 (95% CI: 0.08–0.66), 56–67 mg/dL: OR = 0.11 (95% CI: 0.04–0.35), >67 mg/dL: OR = 0.11 (95% CI: 0.04–0.35).

In unadjusted analysis, RC in the highest quartile (> 28 mg/dL) was significantly associated with cardiovascular events (OR = 2.94; 95% CI: 1.18–7.34; *p* = 0.021), as were triglycerides > 127 mg/dL. HDL cholesterol demonstrated a protective, dose‐dependent effect (Figure 2).

### 3.4 Multivariate analysis

Following unadjusted analysis of lipid fractions and their impact on cardiovascular disease, conditional logistic regression models for matched data were conducted with variables that met confounding criteria. The confound analysis identified nephropathy (11.3% change), insulin/kg (13.1%), BMI (20.7%) and high‐intensity statin therapy (12.0%) as confounders and these variables were retained in the multivariable models. In contrast, no lipid‐lowering therapy reached the predefined threshold and was therefore not considered a confounder in the final analyses.

**FIGURE 3:**
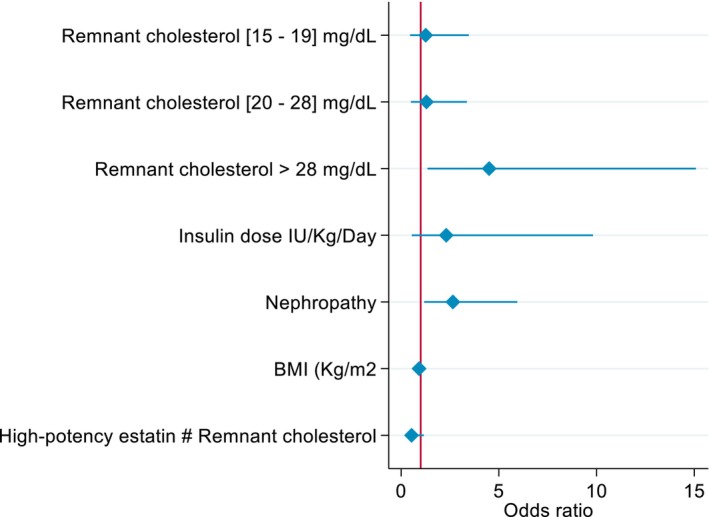
Association of remnant cholesterol quartiles in cardiovascular disease. High‐potency lipid‐lowering treatment is not shown in the graph because the large size of its confidence interval artefacts the proportions of the other variables, and they are not easily visible. Remnant cholesterol values are compared against the reference <15 mg/dL. High‐potency statin—remnant cholesterol indicates the interaction between high‐potency statin therapy and remnant cholesterol in relation to cardiovascular disease. The figure shows that the highest quartile of remnant cholesterol (>28 mg/dL) is independently associated with cardiovascular disease, accounting for other variables and confounding factors.

In the adjusted models, remnant cholesterol in the highest quartile (> 28 mg/dL) remained independently associated with cardiovascular events (OR = 4.50; 95% CI: 1.34–15.08; *p* = 0.015). High‐intensity statin therapy was also associated with increased cardiovascular risk (OR = 11.54; 95% CI: 1.30–102.41; *p* = 0.028), which likely reflects indication bias, as these agents are typically prescribed to individuals with higher baseline cardiovascular risk. Similarly, diabetic nephropathy was independently associated with cardiovascular events (OR = 2.65; 95% CI: 1.18–5.94; *p* = 0.018). BMI showed a borderline inverse association with cardiovascular events (OR = 0.92; 95% CI: 0.84–1.01; *p* = 0.073). No significant interaction was observed between high‐intensity statin therapy and remnant cholesterol quartiles (p for interaction = 0.113) (Figure 3).

**FIGURE 4:**
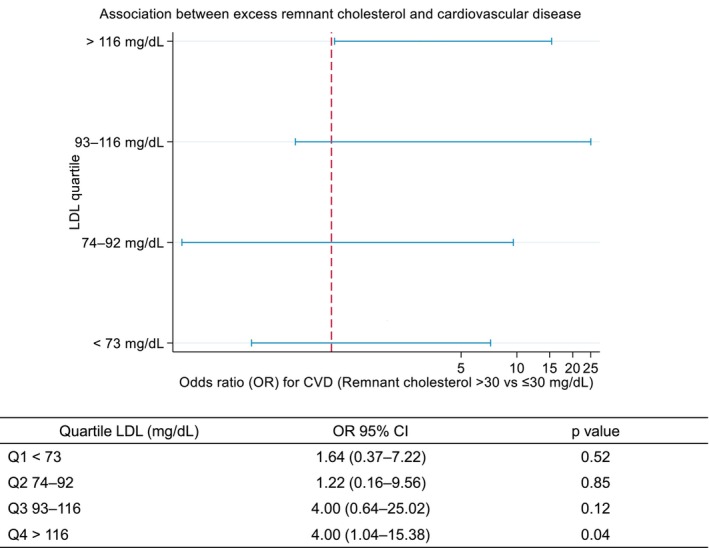
Stratified analysis of the association between excess remnant cholesterol (≥ 30 vs. < 30 mg/dL) and cardiovascular disease risk according to LDL cholesterol quartiles. Odds ratios (ORs) and 95% confidence intervals are shown for each LDL quartile.

To explore potential effect modification, we conducted a stratified analysis evaluating the association between excess remnant cholesterol (defined as > 30 mg/dL) and cardiovascular disease across LDL quartiles. The OR associated with excess remnant cholesterol varied across LDL strata: OR = 1.64 (95% CI: 0.37–7.22) for LDL <73 mg/dL, OR = 1.22 (0.16–9.56) for LDL 74–92 mg/dL, OR = 4.00 (0.64–25.0) for LDL 93–116 mg/dL and OR = 4.00 (1.04–15.4) for LDL >116 mg/dL. The Mantel‐Haenszel test for linear trend was statistically significant (*p* = 0.0197), supporting a potential dose–response relationship (Figure 4).

Finally, as a sensitivity analysis, we repeated the conditional logistic regression models within strata of LDL concentration. Among individuals with LDL <100 mg/dL, no significant association was found between remnant cholesterol quartiles and cardiovascular risk, even in the highest quartile (> 28 mg/dL: OR = 1.24; 95% CI: 0.31–4.94; *p* = 0.763). In contrast, among individuals with LDL ≥ 100 mg/dL, remnant cholesterol in the highest quartile was strongly associated with cardiovascular events (OR = 30.0; 95% CI: 2.77–326.0; *p* = 0.005), independent of statin therapy, insulin dose and nephropathy (see Supporting Information S1).

In multivariable analyses, no covariates met the confounding criteria for HDL cholesterol and therefore no adjusted models were required. HDL cholesterol levels above 45 mg/dL were consistently associated with a reduced risk of cardiovascular disease, while HDL <45 mg/dL conferred a significantly increased risk (OR = 8.84; 95% CI: 2.86–27.3; *p* < 0.001). In contrast, after adjustment for nephropathy, insulin/kg and BMI, triglyceride levels in the highest quartile (>127 mg/dL) remained independently associated with cardiovascular events (OR = 3.16; 95% CI: 1.16–8.58; *p* = 0.024), supporting their close relationship with remnant cholesterol as previously noted.

## 4 Discussion

In this work, we evaluate the impact of remnant cholesterol on the occurrence of established and symptomatic CVD in patients with T1D. Our findings indicate that elevated remnant cholesterol levels are independently associated with an increased risk of CVD and that this association is strongly influenced by LDL cholesterol levels. These results were observed in a multicentre cohort of adults with T1D characterised by suboptimal glycaemic control and high cardiovascular risk and a large proportion of subjects not receiving lipid‐lowering therapy.

Cardiovascular disease remains the leading cause of mortality in people with T1D [4, 29]. Although hyperglycaemia is a key factor associated with adverse cardiovascular outcomes, the relationship between glycaemic control and CVD is not straightforward [30]. Even individuals with good glycaemic control face excess cardiovascular mortality compared to the general population [29, 31], suggesting a need for novel risk markers. Notably, nearly 50% of individuals without baseline CVD in the Pittsburgh Epidemiology of Diabetes Complications Study experienced a cardiovascular event during 25 years of follow‐up [30, 32], pointing to a persistent residual risk potentially driven by nontraditional lipid parameters [28, 33].

Lipid abnormalities may partially account for this risk in T1D [25, 34]. Our findings show that elevated remnant cholesterol is independently associated with cardiovascular events and appears to exert an additive effect in the presence of elevated LDL cholesterol, regardless of glycaemic control, age, nephropathy, smoking status, hypertension or lipid‐lowering therapy. Specifically, the impact of remnant cholesterol was negligible in individuals with LDL < 100 mg/dL, whereas the association was markedly stronger in those with LDL ≥ 100 mg/dL. The sensitivity analysis confirmed a consistent increase in risk across LDL quartiles. Mechanistically, remnant cholesterol represents the cholesterol content in triglyceride‐rich lipoproteins, specifically in VLDL and IDL in a fasting state [35]. Once these particles reach the arterial intima, they are engulfed by macrophages, leading to foam cell formation and promoting atherosclerosis [35]. Furthermore, triglyceride breakdown within these particles releases free fatty acids and monoacylglycerides, which trigger an inflammatory response [36]. Impaired remnant lipoprotein metabolism, along with these particles' higher affinity for proteoglycans, may contribute to arterial cholesterol deposition in people with T1D [37] and are now considered predictors of retinopathy, nephropathy and diabetic foot disease [25, 38]. Although Mendelian randomisation studies suggest a causal role of remnant cholesterol in [8, 10] atherosclerosis, some randomised trials aiming to lower remnant cholesterol have not consistently demonstrated cardiovascular benefit [39]. This may indicate that therapeutic strategies must target both apoB‐containing lipoproteins (i.e., remnant and LDL particles) and the overall burden of circulating apoB particles to achieve effective cardiovascular risk reduction [40].

Our study also found that the association between remnant cholesterol and cardiovascular disease was independent of diabetic nephropathy, although the two were correlated. This is consistent with prior reports linking remnant lipoproteins to albuminuria and kidney disease in diabetes [41, 42], suggesting that remnant cholesterol may contribute to both macrovascular and microvascular complications in T1D. Further prospective studies are warranted to clarify this relationship. Triglyceride levels were associated with cardiovascular disease, which may reflect underlying insulin [43] resistance. This is particularly relevant, as insulin resistance has been consistently identified as a risk factor for subclinical atherosclerosis [44, 45]. In this regard, our study may generate hypotheses along this pathway; however, we lacked validated measures of insulin resistance, such as the estimated glucose disposal rate. In contrast, low HDL cholesterol (< 45 mg/dL) remained strongly associated with increased cardiovascular risk in multivariable models. These findings align with previous studies in T1D, where HDL cholesterol consistently demonstrated a protective role [34]. However, pharmacological strategies to raise HDL levels have not proven effective in reducing cardiovascular events, indicating that HDL‐targeted therapies require further investigation. Finally, we observed that nearly one‐third of cases and over half of controls were not receiving any lipid‐lowering therapy at the time of their cardiovascular event, despite being at high cardiovascular risk. This under‐treatment pattern is consistent with other national studies [46], despite current clinical guidelines recommending similar lipid management strategies for T1D and type 2 diabetes [47]. The low rate of lipid‐lowering therapy use, together with the large gap from LDL targets observed in this study, represents one of its most clinically relevant implications, highlighting the urgent need to achieve LDL goals in order to mitigate the multiplicative cardiovascular risk posed by additional factors such as remnant cholesterol. This study has several limitations. First, the nested case–control design precludes causal inference, limiting conclusions to hypothesis generation. Second, only individuals who survived the cardiovascular event were included in the analysis, which may introduce survivor bias. Third, we did not measure lipid microparticles, inflammatory biomarkers or markers of subclinical atherosclerosis, which may influence cardiovascular risk in T1D [13, 48, 49].

Fourth, we did not apply validated cardiovascular risk prediction tools such as the 2019 ESC/EASD risk stratification algorithm or the Steno Type 1 Risk Engine, which may have provided additional context for individual risk [50] profiles. Fifth, there were between‐group differences in the management of nephropathy and lipid‐lowering therapy that may have introduced residual confounding. Sixth, our cohort had a predominance of coronary artery disease compared to stroke or peripheral artery disease, potentially biasing results toward risk factors specifically associated with coronary ischaemia [34]. We also did not accurately estimate insulin resistance using the estimated glucose disposal rate. Finally, although the association between elevated remnant cholesterol and cardiovascular disease was particularly pronounced in individuals with LDL‐C ≥ 100 mg/dL, the wide confidence interval suggests imprecision and potential overestimation due to the small sample size within this subgroup. These findings should therefore be interpreted cautiously and warrant confirmation in larger prospective cohorts.

## 5 Conclusions

Elevated remnant cholesterol levels are independently associated with an increased risk of cardiovascular disease in individuals with T1D, particularly among those with LDL cholesterol ≥ 100 mg/dL. These findings support the hypothesis that remnant cholesterol contributes to residual cardiovascular risk beyond traditional lipid markers. Achieving LDL targets is essential to minimise the impact of other lipid abnormalities on cardiovascular risk. Further large‐scale, prospective studies are warranted to validate these observations and to guide the development of targeted lipid‐lowering strategies in this high‐risk population.

## Author Contributions

Fernando Sebastian‐Valles: Writing – original draft, Writing – review and editing, Supervision, Project administration, Conceptualization, Methodology. Iñigo Hernando‐Alday: Writing – review and editing, Formal analysis, Data curation. Luis Eduardo Lander Lobariñas: Resources, Investigation, Data curation. Maria Luisa Palacios Berraquero: review and editing, Investigation. Jon Garai‐Hierro: Resources, Investigation. Gisela Liz Roman‐Gomez: Resources, Investigation, Visualization. Álvaro Montes Muñiz: Writing – review and editing, Formal analysis. Victor Navas‐Moreno: Writing – review and editing, Resources, Investigation. Purificación Martinez de Icaya: Writing – review and editing, Data curation. Juan José Raposo‐López: Resources. Miguel Antonio Sampedro‐Nuñez: Writing – review and editing, Supervision. Carmen González‐Ávila: Writing – review and editing, Investigation. Jose Alfonso Arranz‐Martín: Writing – review and editing, Supervision, Conceptualization. Mónica Marazuela: Writing – review and editing, Supervision, Project administration, Conceptualization, Methodology.

## Conflicts of Interest

The authors declare no Conflicts of Interest.

## Supporting information


**Supporting Information S1.** Sensitivity Analysis by LDL Level

## Data Availability

The data that support the findings of this study are available from the corresponding author upon reasonable request.
