# Supplementary material for: Remnant Cholesterol and Cardiovascular Risk in Adults With Type 1 Diabetes: A Nested Case–Control Study
Source: Endocrinol Diabetes Metab. 2025 Oct 8;8(6):e70114. doi: 10.1002/edm2.70114 (PMC12504852; doi:10.1002/edm2.70114)
Supplement: Supplementary file 1 — Supporting Information S1. Sensitivity Analysis by LDL Level [file EDM2-8-e70114-s001.docx]

# Supplementary Material S1. Sensitivity Analysis by LDL Level

## LDL ≥100 mg/dL (n=68)

| Variable | Odds Ratio | 95% CI | p-value |
| --- | --- | --- | --- |
| ColRem 15–19 mg/dL | 1.83 | 0.21–15.71 | 0.582 |
| ColRem 20–28 mg/dL | 1.63 | 0.23–11.73 | 0.629 |
| ColRem >28 mg/dL | 30.05 | 2.77–326.04 | 0.005 |
| Insulin/kg | 61.50 | 1.91–1983.26 | 0.020 |
| Nephropathy | 2.30 | 0.53–9.99 | 0.265 |
| BMI | 0.91 | 0.76–1.10 | 0.328 |
| High-potency statin | 118.84 | 0.56–25268.78 | 0.081 |
| Interaction (high-potency statin × ColRem) | 0.18 | 0.03–1.05 | 0.057 |

## LDL <100 mg/dL (n=108)

| Variable | Odds Ratio | 95% CI | p-value |
| --- | --- | --- | --- |
| ColRem 15–19 mg/dL | 0.75 | 0.24–2.33 | 0.613 |
| ColRem 20–28 mg/dL | 0.80 | 0.24–2.69 | 0.723 |
| ColRem >28 mg/dL | 1.24 | 0.31–4.94 | 0.763 |
| Insulin/kg | 1.26 | 0.27–5.82 | 0.770 |
| Nephropathy | 2.55 | 0.95–6.86 | 0.063 |
| BMI | 0.93 | 0.83–1.03 | 0.141 |
| High-potency statin | 3.33 | 0.30–37.41 | 0.329 |
| Interaction (high-potency statin × ColRem) | 0.99 | 0.42–2.33 | 0.973 |
